# Layout Design of Human-Machine Interaction Interface of Cabin Based on Cognitive Ergonomics and GA-ACA

**DOI:** 10.1155/2016/1032139

**Published:** 2016-01-18

**Authors:** Li Deng, Guohua Wang, Suihuai Yu

**Affiliations:** ^1^School of Mechatronic Engineering, Southwest Petroleum University, Chengdu 610500, China; ^2^Institute of Industrial Design, Northwestern Polytechnical University, Xi'an 710072, China; ^3^State Key Laboratory of Oil and Gas Reservoir Geology and Exploitation, Southwest Petroleum University, Chengdu 610500, China

## Abstract

In order to consider the psychological cognitive characteristics affecting operating comfort and realize the automatic layout design, cognitive ergonomics and GA-ACA (genetic algorithm and ant colony algorithm) were introduced into the layout design of human-machine interaction interface. First, from the perspective of cognitive psychology, according to the information processing process, the cognitive model of human-machine interaction interface was established. Then, the human cognitive characteristics were analyzed, and the layout principles of human-machine interaction interface were summarized as the constraints in layout design. Again, the expression form of fitness function, pheromone, and heuristic information for the layout optimization of cabin was studied. The layout design model of human-machine interaction interface was established based on GA-ACA. At last, a layout design system was developed based on this model. For validation, the human-machine interaction interface layout design of drilling rig control room was taken as an example, and the optimization result showed the feasibility and effectiveness of the proposed method.

## 1. Introduction

Human-machine interaction interface is the medium between human and machine to transmit information and is the specific form of expression between the human, machine, and environment, which is also the necessary means to realize the interaction. In the model of human-machine system, through the visual and auditory organ, people receive information from machine; then, with the processing and decision in brain, the locomotive organ reacts to realize the information transmission between human and machine. On the other hand, all sorts of machine's display have effect on people, which will realize the information transmission between machine and human.

Cha et al. [1] proposed that layout design problem was an important part of the design field. Layout design problem is to put objects in space reasonably, to meet the necessary constraints and achieve some kind of optimal index [2]. The layout problem involves bin packing problem, strip packing problem, cutting stock problem, office layout design [3], workshop layout design [4], and so on. At present, the solving approach of layout problem is usually to simplify the practical engineering problem into mathematical model and then through computer algorithm to solve. For example, Huo et al. [5] presented human-machine cooperation immune algorithm, ant colony algorithm, genetic algorithm, and other methods to solve the problem of satellite cabin layout design and acquired the engineering satisfactory solution and superior computational efficiency. Li et al. [6] put forward the aircraft cockpit layout optimization design method, including the layout of display and operating device. Yan et al. [7] put forward the optimization method of controls layout based on simulated annealing algorithm. Zong et al. [8, 9] established the layout optimization mathematical model of manned submersibles cabin, proposed multiobjective optimization calculation method based on the Pareto PGA algorithm, and proposed artificial fish algorithm to solve the deep submergence cabin layout optimization problem. Wang et al. [10] established layout optimization mathematical model of ship cabin based on improved genetic algorithm.

The layout problem of human-machine interaction interface was getting more and more inseparable with artificial intelligence technology. Researchers began to pay attention to various computational intelligence algorithms, such as genetic algorithm, particle swarm optimization algorithm, simulated annealing algorithm, ant colony algorithm, and tabu search. Besides, according to the NO Free Lunch theorem proposed by Professors Wolpert and Macready in Stanford University [11], a single optimization algorithm had its advantages and disadvantages of application. From the perspective of solving optimization problem, the fusion of different types of algorithmic mechanism and giving full play to their respective advantages were the inevitable development trend to solve the problem. Hybrid intelligent optimization algorithm had become an important strategy to solve practical engineering problems [12].

The effect of the algorithm in different applications was different, so, according to the characteristics of the cabin, solving algorithm should be suitable for the layout problem. The current layout research was focused on the improvement of space utilization [13], so the traditional optimization objective was difficult to meet the requirements of human body's comfort in operating. Human physiological and psychological characteristics should be considered in the layout design, so as to make the operators have comfortable operating posture. Thus, from the perspective of cognitive psychology [14], the layout principles of human-machine interaction interface were summarized, and the human cognitive characteristics were quantified as the layout constraints. GA-ACA [15] would be applied to realize the intelligent layout optimization of human-machine interaction interface of cabin.

## 2. Implementation Method

### 2.1. Outline of the Proposed Method

This paper defined that the cabin was a kind of semiclosed or totally enclosed space, mainly including aerospace manned cockpit, engineering machinery cab, automobile cab, submersible manned cabin, oil rig driller control room, and nuclear power plant control room. Through human-machine interaction interface, the operators proceeded with operation tasks in the interior space of cabin. As the operators' working environment, the cabin directly impacted on the operators' working status. Reasonable layout design of human-machine interaction interface could improve the operators' identification ability and work efficiency. On the contrary, it was possible that improper layout design can lead to wrong operation and occupational disease and even affect the safety of the operating system.

American cognitive psychologist Neisser defined that cognitive psychology is to study all mental processes by which the sensory input is transformed, reduced, elaborated, stored, recovered, and used [16]. From the perspective of cognitive ergonomics, human-machine interaction process was a cognitive process. Interactive process was not only the feeling process of the physiological action and the stimulus signal, but also one kind of information processing process. The human-machine interaction interface layout design should adapt to people's understanding and operating process.

**Figure 1:**
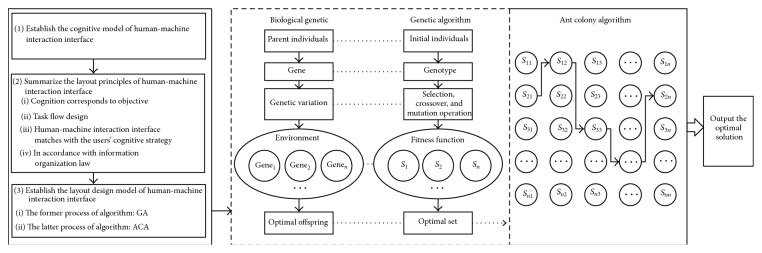
The implementation steps.

The implementation steps of layout design method are shown in Figure 1.

**Step 1 (establish the cognitive model of human-machine interaction interface).**

The reasons of cognitive errors could be analyzed by the cognitive model, which described the cognitive process. Taking the improvement of cognitive ergonomics as the goal, the cognitive theory was used to guide the layout design of human-machine interaction interface.

**Step 2 (summarize the layout principles of human-machine interaction interface).**

Adhering to the idea of “User Centered Design,” users were fully considered in the layout design. According to the analysis of cognitive ergonomics, the layout principles of human-machine interaction interface were summarized on the basis of human cognitive characteristics.

**Step 3 (establish the layout design model of human-machine interaction interface).**

Hybrid intelligent optimization algorithm was used to solve the combination optimal problem. The layout design model of human-machine interaction interface was established based on GA-ACA, which combined the advantages of GA and ACA.

### 2.2. Establish the Cognitive Model of Human-Machine Interaction Interface

Human-machine interaction process is actually a process of information processing. Through the characteristics about mental labor in cognitive psychology, such as the research of memory, understanding, and communication, the designed human-machine interaction interface tries to reduce people's cognitive burden as much as possible, making the product easy to learn, easy to use, and with high efficiency. The idea of human-machine interaction interface layout design was to build cognitive model based on the information processing mechanism. Then, on the basis of people's thinking characteristics, using the rule of information organization, visual search pattern, and memory characteristic, the human-machine interaction interface layout would conform to operators' cognitive ability for interface information.

**Figure 2:**
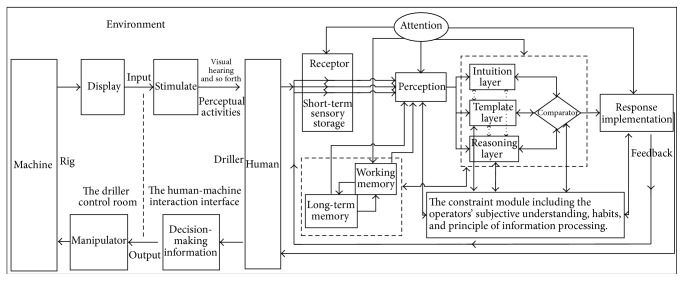
The cognitive model of human-machine interaction interface of cabin.

There were plenty of studies on cognitive processes; psychologists' emphasis on the study of cognitive processes was different at different periods. Information processing model described the main elements or stages in the human information processing and the hypothetical relationship between them. Most of the models were consistent with this basic framework. And on this basis Wickens et al. [17] put forward the model of information processing with the attention function. Sun et al. [18] put forward cognitive synthetic model, which brought cognitive process into the interaction between human and environment system, according to the thought of parallel processing of layered information, and output by competition and coordination. Taking and integrating the advantages of the predecessor's research as reference, this paper put forward the cognitive model of human-machine interaction interface of cabin (shown in Figure 2).

As can be seen from Figure 2, cognitive processes are placed in the interaction system of human, machine, and environment. Through visual and auditory organ, the operators observe the system's operating situation and proceed with sensory processing. Combining call rules and knowledge in long-term memory and call targets and tasks in short-term memory with constraint module, the sensory information processing is conducted. Information processing includes intuition layer, template layer, reasoning layer, and comparator. Information is parallel-processed by the three layers of intuition, template, and reasoning, and the schemes are produced in the competition and cooperation in the three layers and output in the comparator. Finally, the selected corresponding method is carried out. Information acquisition, processing, and performing all need to interact with the constraint module, which is the operators' subjective understanding, habits, and principle of information processing. Perception, decision-making, and response implementation all need to consume attention. In order to obtain the best cognitive ergonomics, appropriate cognitive strategies should be adopted to balance the quality and speed of the information processing in cognitive system. This model could describe the operators' cognitive process clearly, so it is suitable for application in the layout design of human-machine interaction interface of cabin.

### 2.3. Summarize the Layout Principles Based on Cognitive Psychology

The information processing is not only affected by stimulation but also influenced by the past experience and knowledge. To improve the efficiency of human-machine interactive information, cognitive law involving the human-machine interaction interface layout design should be summarized, which would be transformed into guiding principles for layout design [19, 20].

**Principle 1 (cognition corresponds to objective).**

Using the human-machine interaction interface in line with users' experience and knowledge and adopting the appropriate processing method conform to the old habits and concepts, which could reduce the learning time and memory time and avoid the happening of mistakes. For instance, the manipulators with similar functions should be arranged in the same area. Because when the manipulators with similar functions needed to be used, the attention of operators would search the manipulator at the familiar area of interface in an automatic way.

**Principle 2 (task flow design).**

Through the operating sequence design to reduce the users' workload and improve the working efficiency, in view of the main tasks of the operator that need to be done, the execution process of tasks should be analyzed. And then according to operators' cognitive habits to merge or reduce the unnecessary actions or implement automation, so as to simplify the dialogue process of human-machine interaction, which would speed up the information processing and reduce the users' cognitive load and cognitive time in information processing.

**Principle 3 (human-machine interaction interface matches with the users' cognitive strategy).**

Human cognition had dynamic characteristic: the human-machine interaction interface was very difficult to adapt to the dynamic cognitive differences of all kinds of users. So, based on users' knowledge level, cognitive ability, and habits, meanwhile, by judging the different levels of users' cognitive strategy, human-machine interaction interface would be designed intelligently, so as to adapt to the users' cognitive process dynamically. For example, according to the characteristics of memory, the important display and manipulator, which would be frequently observed and manipulated, should be arranged in the convenient range for observation and comfortable range for manipulation. The burden on the users' short-term memory could be eased. And it was advantageous to reasonably distribute cognitive load between layers of intuition, template, and reasoning and avoiding forgetting and memory errors such as consequence.

**Principle 4 (in accordance with information organization law).**

The discrete stimulate in view could be organized together and formed the vision of a whole, by the certain relationship between them, and this phenomenon was known as the visual organization features. Visual organizational principles mainly included proximity principle, similarity principle, and closeness principle, which had a certain guiding significance in the interface design. For example, suppose there was lots of information that needed to be displayed on the screen; in order to make the information displayed clearly, the relevant information should be put together by proximity principle or be expressed in the same color by similarity principle. So this information seemed to be whole, and the users could detect them rapidly and accurately.

### 2.4. Determine the Optimization Objective according to the Layout Principles

**Figure 3:**
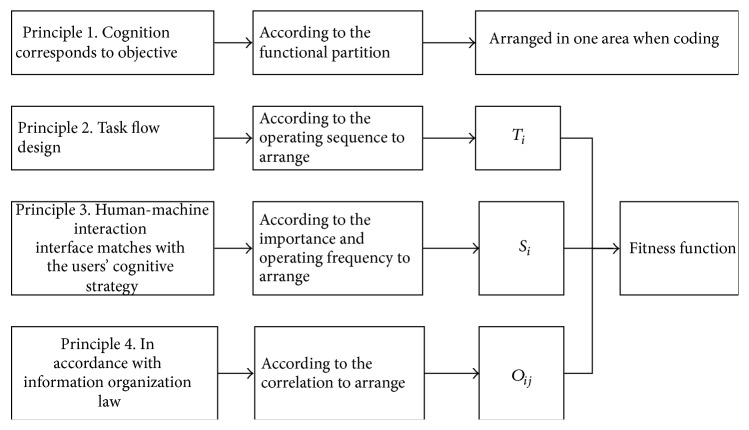
The process of building objective function.

The human-machine interaction interface layout problem could be transformed into the combinatorial optimization problem; that is, the layout scheme which most met the layout principles was sought from different permutation and combination scheme of the layout objects. The optimization objective was to find the best layout scheme, making the objective function get the maximum value. The process of building objective function is shown in Figure 3.

In short, layout Principle 1 embodied in the layout objects with similar function will be arranged in one area. Layout Principle 2 is embodied in the arrangement in accordance with the physical activities characteristics from left to right and from top to bottom. Layout Principle 3 embodied in the important layout objects will be arranged near the best layout point *k*. Layout Principle 4 embodied in the related layout objects will be arranged close to each other.

According to the layout principles, the objective function is defined as follows:

(1) fibest=arg⁡max⁡∑i=1nδ1·Ti+∑i=1nδ2·Si+∑i=1n ∑j=i+1nOijdij,

(2) δ1=1−in,

(3) δ2=1−dikdkm,

where *T*_*i*_ represents the weight of layout object *i* relative to Principle 2. *S*_*i*_ represents the weight of layout object *i* relative to Principle 3. *O*_*ij*_ represents the correlation between layout object *i* and layout object *j* relative to Principle 4. *d*_*ij*_ represents the distance between layout object *i* and layout object *j*. *n* represents the number of layout objects. *i* represents the position number of layout objects. *δ*_1_ and *δ*_2_ represent the position control coefficients. *d*_*ik*_ represents the distance between the layout object *i* and the best layout point *k*. *d*_*km*_ represents the maximum distance between a layout point and the best layout point *k* in the whole layout range.

### 2.5. Establish the Layout Design Model of Human-Machine Interaction Interface Based on GA-ACA

#### 2.5.1. GA-ACA

In 1975, according to Darwin's theory of survival of the fittest, the American scholar John Holland put forward the genetic algorithm [21]. Through parallel and global search mode to search the best individual in the optimization group, GA had good adaptive ability, robustness, generality, and other merits, widely used in machine learning, pattern recognition, image processing, and other fields.

In the early 1990s, inspired from ants' foraging behavior in the nature world, the Italian scholar Dorigo et al. presented ant colony algorithm [22–24]. ACA had very strong robustness and ability to search a good solution and easy to parallel implementation. Originally it is used to solve the traveling salesman problem, latter widely applied to solve the classical optimization problem, such as sequential ordering problem and quadratic assignment problem.

GA and ACA were stochastic optimization algorithm, both of which had the advantages of global search and random search, and easily combined with other algorithms. However, GA had the shortcomings of huge calculation and poor stability, leading to low precision and efficiency. ACA needed long search time and is easily prone to stagnation phenomenon. The combination of GA and ACA could utilize the advantage of the two algorithms and overcome their disadvantages, and the research has proved that the hybrid algorithm had good efficiency [25]. This new algorithm was used for multiple sequence alignment [26], initialization for synchronous sequential circuits [27], hardware/software partitioning [28], and other optimization problems.

**Figure 4:**
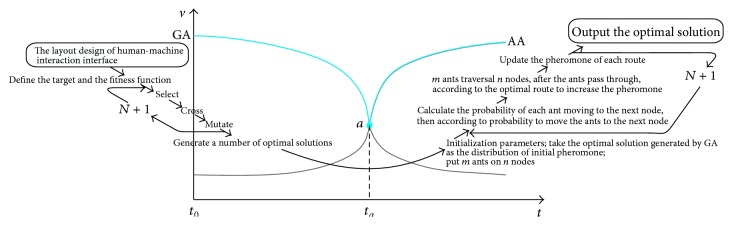
The process of GA-ACA.

According to the study and experiment of GA and ACA [29], the speed-time curve is shown in Figure 4. In the early stages of the search (*t*_0_ ~ *t*_*a*_), GA has fast convergence. When evolution reaches a certain degree, its evolutionary rate would fall sharply; namely, the efficiency is low after *t*_*a*_. Instead, at the beginning of the search (*t*_0_ ~ *t*_*a*_), ACA searches slowly. But after the accumulation of pheromone reaches a certain extent, the searching activity of ants can present a certain regularity; that is to say, the speed is rapidly improved after *t*_*a*_.

As shown in Figure 4, this paper put forward combining the advantages of the two algorithms in layout design. GA is adopted in the former process of algorithm (before point *a*), and the rapidity, randomness, and global convergence of GA are used. A number of layout schemes of human-machine interaction interface are generated as initial solution, which will be turned into track intensity distribution as initialization pheromone. Then, ACA is adopted to optimize the layout schemes in the latter process of algorithm (after point *a*). In the case of a certain track intensity distribution of initialization pheromone, the characteristics of parallelism, positive feedback mechanism, and high solving efficiency will be used to improve the efficiency of solving and output the optimal solution.

#### 2.5.2. GA to Determine the Initial Pheromone

(*1) The Operating Mechanism of GA*. The process of GA in human-machine interaction interface layout is shown at the left part in Figure 4.

*(a) Coding*. Using the form of sequence coding, the layout objects were expressed by real number to carry out optimizing calculation. The code string format shows as follows:

(4) $$\begin{array}{c} C_{m}=\left[\;c_{1}^{m},c_{2}^{m},c_{3}^{m},\ldots ,c_{n}^{m}\;\right], \end{array}$$

where *m* represents the population size. *n* represents the number of the layout objects. *c*_*n*_^*m*^ represents the layout object in the *n*th position of the *m*th chromosome.

Suppose there are 10 layout objects, which randomly generated an individual, such as [5,4, 3,8, 7,2, 10,1, 9,6]. The code string represents layout object 5 in the first position, layout object 4 in the second position, and so on. There are direct relationships between the objective function and the coordinate of layout objects. If the codes of layout objects are in different position, the corresponding coordinates are different, and the objective function value will also be different. 

*(b) Construct the Fitness Function*. Take each kind of permutation and combination way as an individual, and then the best layout solution was sought by calculating the individual's fitness value and evolutionary operation. The *p*th fitness value of chromosome is as follows:

(5) $$\begin{array}{c} \text{fitness}\left(\;p\;\right)=\overset{n}{\underset{i=1}{\sum }}\delta_{1}\cdot T_{i}+\overset{n}{\underset{i=1}{\sum }}\delta_{2}\cdot S_{i}+\overset{n}{\underset{i=1}{\sum }}\;\overset{n}{\underset{j=i+1}{\sum }}\frac{O_{ij}}{d_{ij}}. \end{array}$$

The three parts of fitness function reflect the comprehensive situation of layout Principles 2, 3, and 4, respectively; the pros and cons of layout schemes will be judged from the overall layout.

*(c) Determine the Evolutionary Mechanism*. It is done starting from the random generation of initial population, constantly repeating the process of selection, crossover, and mutation operation, and the population developed along the direction of established goals from generation to generation. According to the fitness value of the fitness function, excellent individuals would be selected from the current population by wheel bet method, so as to form a new population. Using sequence crossover method, parent individuals exchanged part of genes, and thus new individuals would be formed. Using swap mutation method, the diversity of population was guaranteed, and immature convergence phenomenon was prevented.

(*2) The Combination of GA and ACA*. The combination opportunity of GA and ACA was dynamically determined in the operational process of GA. First set minimum genetic iterations Gen_min_ and maximum genetic iterations Gen_max_ in GA. Then, according to formula (6), record the evolution rate of progeny population in the iterative process, and set the minimum evolution rate Gen_*p*_ of progeny population. Within the scope of the given number of iterations, if successive Gen_*q*_ generations, the evolution rate of progeny population, were less than Gen_*p*_, this showed that the optimization speed was very low. The process of GA should be terminated and turned into the ACA. The 10% of the optimal individuals in last generation in GA would be selected as the carrier, which would be taken as the suboptimal solution to distribute the initial pheromone in ACA, and optimal solution would be obtained further:

(6) $$\begin{array}{c} {EV}^{k}=\frac{\sum_{i=1}^{m}\left(f_{i}^{k}-f_{i}^{k-1}\right)/f_{i}^{k-1}}{m},\;k=2,3,\ldots ,n, \end{array}$$

where *m* represents population size. *k* represents evolutional generation. *f*_*i*_^*k*^ represents the fitness value of individual *i* in *k*th iteration.

#### 2.5.3. ACA to Solve Pareto Solution

The purpose of layout optimization of human-machine interaction interface was to get an optimal solution to satisfy the design goal, which was to find an optimal path from layout object *i* to layout object *j*, and the optimized goal was to get a maximum of (1). In the ACA system model, each feasible solution represented a path that an ant walked by. It is a shortest path problem, so, the objective function of ACA was defined as the reciprocal of the objective function of GA:

(7) $$\begin{array}{c} L_{k}=\frac{1}{f{\left(i\right)}_{best}}. \end{array}$$

(*1) State Transition Rules*. The process of ACA in human-machine interaction interface layout is shown at the right part in Figure 4. Take the layout of manipulators to describe the realization process of ACA [30, 31]. Set *b*_*i*_(*t*)  (*i* = 1,2,…, *n*) represents the number of ants of manipulator *i* in time *t*, *m* = ∑_*i*=1_^*n*^ *b*_*i*_(*t*) represents the total number of ants, and *n* represents the number of manipulators. Set tabu_*k*_ represents the tabu table of ant *k*, which will be adjusted dynamically along with the ant optimization process. In initial stage, *m* ants will be placed on *n* manipulators randomly. The first element of each ant's tabu table will be set as its first manipulator. In the process of each iteration, each ant chooses the next manipulator by the state transition rules repeatedly. After selection for *n* − 1 times, a group of manipulators layout will be generated eventually. The probability of ant *k* transfers from manipulator *i* to manipulator *j* in time *t* is as follows:

(8) pijkt=τijtαηijβ∑l∈Uτiltαηilβj∈U,0j∉U,ηij=1vij,vij=Ti−Tj+Si−Sj+Oi−Oj,

where *τ*_*ij*_(*t*) represents the pheromone between manipulator *i* and manipulator *j* at *t* moment. *η*_*ij*_ represents the visibility (heuristic information) of transferring from manipulator *i* to manipulator *j*. *v*_*ij*_ represents the difference value of comprehensive weight between manipulator *i* and manipulator *j* to the layout principles. *α* (*α* ≥ 0) represents the relative importance of *τ*. *β* (*β* ≥ 0) represents the relative importance of *η*. *U* represents the feasible point set; namely, the set of manipulators can be chosen by ant *k* at *t* moment.

(*2) Pheromone and Heuristic Information*. This paper adopted the max-min ant system (MMAS) which was proposed by the Belgium scholar Thomas. Due to the combined with GA, initialized pheromone was different from MMAS. At the initial moment, initial pheromone value is set as follows:

(9) $$\begin{array}{c} \tau_{ij}\left(\;0\;\right)=\tau_{C}+\tau_{G}, \end{array}$$

where *τ*_*C*_ is the given pheromone constant based on the specific scale of solution, equivalent to the *τ*_min_ in MMAS. *τ*_*G*_ represents the value of pheromone converted from the solution of GA.

Ant chose next manipulator mainly according to pheromone *τ*_*ij*_(*t*) and heuristic information *η*_*ij*_. *η*_*ij*_ was given by the layout problem to be solved and affected by *v*_*ij*_, which remained unchanged during the operation process in algorithm. Ants released pheromone on the path they passed by. In order to avoid too much residual information to submerge heuristic information, residual information should be upgraded. After ants traversed through all the manipulators, the pheromone updated in the environment. The update equation is as follows:

(10) τijt+1=1−ρ·τijt+Δτijt,Δτijt=∑Δτijkt,Δτijkt=QLkant k  passes  though  i,j,0otherwise,

where *ρ*  (0 ≤ *ρ* ≤ 1) represents the volatilization coefficient of pheromone. Δ*τ*_*ij*_^*k*^(*t*) represents the amount of pheromone released between manipulator *i* and manipulator *j* by ant *k* in this cycle. Δ*τ*_*ij*_(*t*) represents the increment of pheromone between manipulator *i* and manipulator *j* after this cycle. *Q* is a constant which represents the amount of pheromone. *L*_*k*_ represents the objective function value corresponding with the layout of all the manipulators formed by ant *k* traversed through in this cycle.

Only the ant with optimal layout scheme could update pheromone in one cycle. The amount of pheromone on each path would be limited in scope of [*τ*_min_, *τ*_max_]. If beyond this range, the pheromone would be mandatory set as *τ*_min_ or *τ*_max_. Compared with the standard ACA, the improved algorithm prevented premature stagnation. With all the ants completing the traverse of all the manipulators, the pheromone accumulated and volatilized continuously. Until reaching the number of iterations or a certain fitness value, the optimal path formed by ants was the best layout scheme.

## 3. Application Verification

### 3.1. Layout Design of Driller Control Room on Drilling Rig

**Figure 5:**
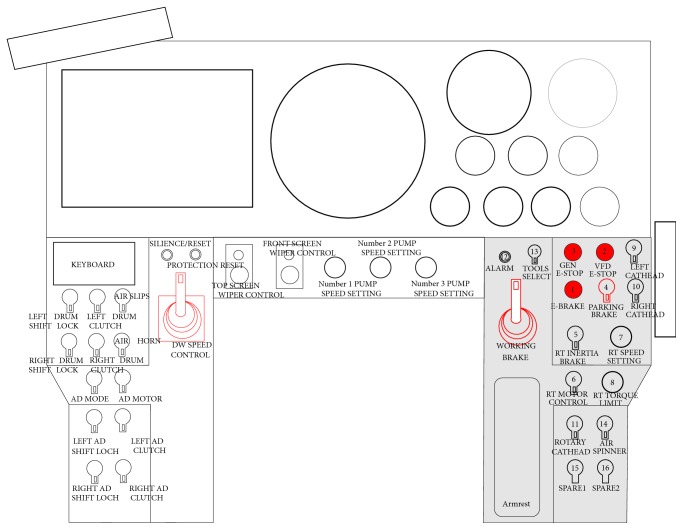
Layout schematic diagram of ZJ120/9000DB rig console (before optimization).

Take the layout design of human-machine interaction interface of driller control room on drilling rig as an example to illustrate the proposed method. The 16 manipulators of ZJ120/9000DB rig console would be arranged (the grey area as shown in Figure 5). First of all, the 16 manipulators need to be encoded. As shown in Table 1, when encoding, layout Principle 1 needs to be reflected; that is, manipulators with similar function will be arranged in one area, to reduce the search time for operators.

It is necessary to simplify the layout area and layout objects when describing the mathematical model, to facilitate a digital description of design variables. According to the human upper limb dimension to determine the layout space and simplify the layout area as rectangular area (450 × 400 mm), there are three types of manipulators, including buttons, rotary knobs, and switch knobs. Their sizes are slightly different, and the specific sizes are shown in Table 2. The spaces between manipulators are set as equal, and the interval is 100 mm. After being simplified, the layout of manipulators can be regarded as a scheduling problem, first using GA-ACA to find a good sorting and then according to the order to establish the actual layout.

Drilling process is mainly divided into three working conditions: pull out of hole, run in hole, and normal drilling. Considering the principle of operating sequence in multiple working conditions, determine the manipulators' operating sequence and calculate each manipulator's weight relative to Principle 2. Through questionnaires to consult drillers, determine the relative judgment matrix of the manipulators' importance and frequency and calculate each manipulator's weight relative to Principle 3. Analytic hierarchy process will be used to calculate each manipulator's weight of *T*_*i*_ and *S*_*i*_, and the calculation result is shown in Table 3. Finally, determine the relative correlation between the manipulators. The correlation between manipulator and itself is expressed with 1, and the correlation between the manipulator and other manipulators is expressed in decimal within 0~1. The greater the correlation between manipulators, the greater the correlation value. The correlation between the 16 manipulators is shown in Table 4.

### 3.2. Optimization Result

**Figure 6:**
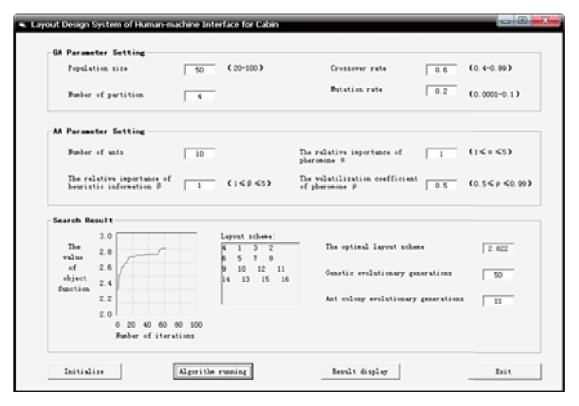
System interface of layout design.

After completing the above data preparation, as shown in Figure 6, the operating parameters of GA and ACA need to be set up before the computer aided optimization calculation proceeds. First, set the control parameters of GA: population size *N* = 50, crossover rate *p*_*c*_ = 0.6, mutation rate *p*_*m*_ = 0.2, and number of partition *s* = 4. Second, set the control parameters of ACA. The amount of ants is *m* = 10, *α* = 1, *β* = 1, and *ρ* = 0.5.

In the system of GA-ACA calculation module, set the end condition of GA: minimum genetic iterations Gen_min_ = 15, maximum genetic iterations Gen_max_ = 50, minimum evolution rate Gene_*p*_ = 3%, and Gene_*q*_ = 3. Set the control parameters of pheromone: *Q* = 500, *τ*_min_ = 10, and *τ*_max_ = 100. The optimal 10% of individuals in the last generation in GA would be selected as the solution set of genetic optimization, which would be transformed into pheromone values. If manipulator *i* was adjacent to manipulator *j* in genetic optimization solution, the pheromone added 10 on the path (*i*, *j*), and all the initial values of pheromones should be set like this. When meeting one of the following conditions, the ACA should stop. (1) The number of iterations reaches ant_max_ = 50. (2) For continuous three generations in the iteration, the improvement rate of offspring optimization is less than 0.5%.

**Figure 7:**
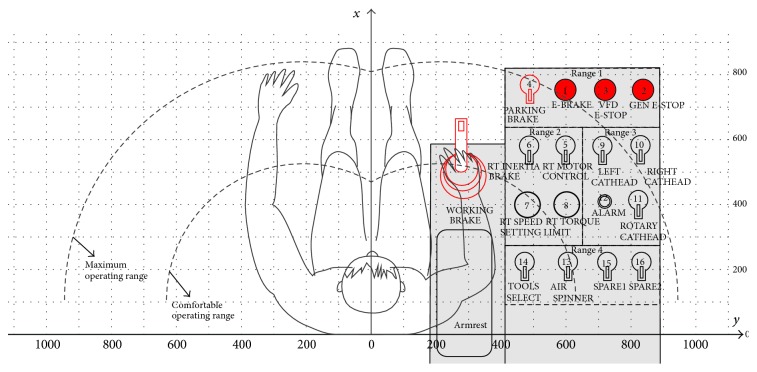
Layout scheme of right console (after optimization).

Click the button of algorithm running on the system interface, the system calculates via the program, after 50 times genetic iterations and 11 times ant colony optimization iteration; the value of the optimal layout scheme is 2.822. The sequence of the manipulators is obtained, that is, 4, 1, 3, 2, 6, 5, 7, 8, 9, 10, 12, 11, 14, 13, 15, and 16. According to the optimized sequence, the layout scheme is shown in Figure 7.

**Figure 8:**
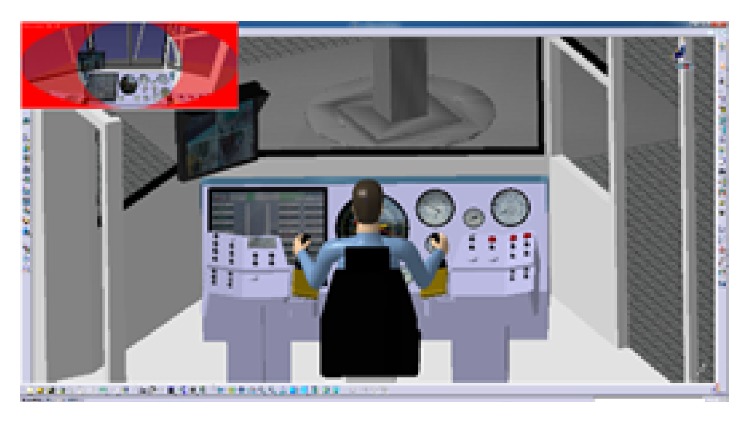
Modeling and simulation of layout scheme.

Because the proposed method is still in the research and development, there is certain difference between the solution and the actual situation. Besides, the human cognitive activities and layout experience cannot be completely described by the layout model; layout scheme obtained by algorithm optimization is close to the optimal solution rather than the optimal solution. In this stage, the designer needs to adjust the layout scheme according to the actual needs. Similarly, sort the manipulators on the console at the left hand. For the sake of evaluating layout scheme intuitively, computer simulation design modeling is proceeded. Through detailed design, the final layout scheme is shown in Figure 8.

**Figure 9:**
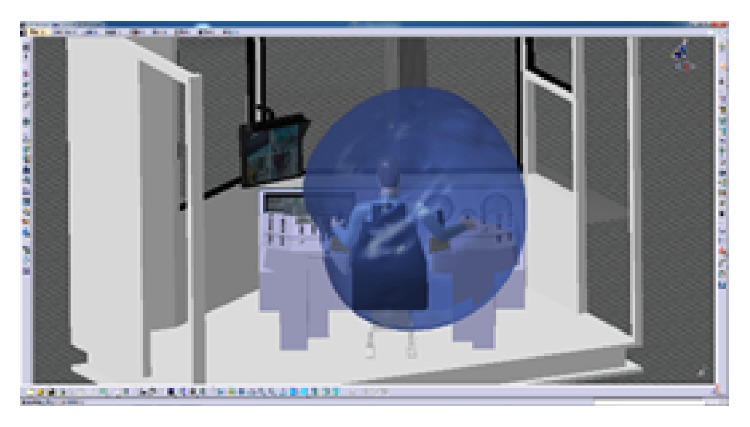
Analyze the right hand's reach envelope.

**Figure 10:**
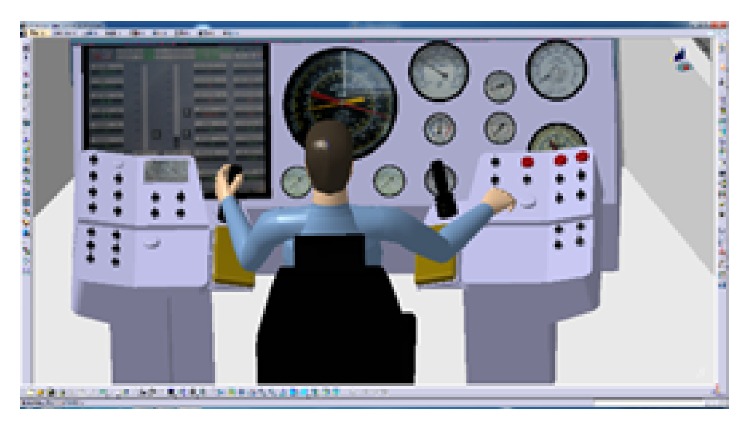
The simulation of operating posture.

**Figure 11:**
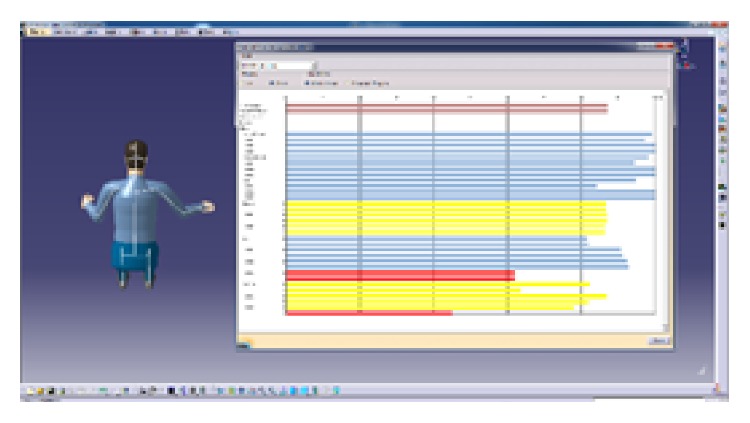
The comfort evaluation of operating posture.

The CATIA software will be used to evaluate the result of the layout. CATIA V5 integrates four ergonomic modules, which can evaluate visibility, accessibility, the comfort of posture (analyze the angle of each joint), and so on. The blue area in Figure 9 shows the reach envelope of driller's right hand. Under natural state, the driller's arm can operate the main manipulators on console. This shows that the locations of manipulators were in the human body's corresponding range of joint motion and in accord with the arm's motion trail. In Figure 10, the virtual human model simulates one operating posture of driller. And the postural score analysis in Figure 11 shows the comfort score of lumbar vertebra, thoracic vertebra, head, arm, and forearm. The sores indicate that this operating posture is comfortable; the position of this manipulator matches with the movement characteristics of the human body.

### 3.3. Algorithm Comparison

Using single GA, ACA, and GA-ACA hybrid algorithm to solve the above layout optimization problem, respectively, set the number of test times as 10 each. Result comparison is shown in Table 5, the column of layout scheme shows the optimal result in 10 times' running, and the third and the fourth columns show the average value of layout scheme and the average number of iterations. As can be seen from the test result, no matter the solution speed or precision, the GA-ACA is superior to single GA and ACA in solving layout optimization problem.

### 3.4. Discussion

Through reference to previous research about cognitive model, this paper integrated their advantages and put forward the cognitive model of human-machine interaction interface suitable for cabin. This model described the cognitive process of operators working in the cabin. And then, by analyzing the information processing process, the layout principles of human-machine interaction interface were summed up, which would be quantified in the objective function. However, human cognitive behavior process is very complicated; part of the cognitive activities is difficult to express by formalized methods; layout principles needed to be improved further. There were lots of influence factors in actual engineering problem; the layout problem was difficult to be described completely by mathematical model.

It has become common that the bionic intelligence algorithms were applied in the layout optimization design, but cognitive characteristics were seldom considered as the constraints of layout optimization algorithm. Furthermore, GA-ACA combined the advantages of two algorithms, which would be more precise to solve layout optimization problem. The layout design of human-machine interaction interface of driller control room on drilling rig has illustrated the proposed method. According to the characteristics of the human-machine interaction interface of different product, this method could be modified and adapted. As a layout ergonomic design method, it could assist designers and engineers to conduct human-machine interaction interface design and improve the efficiency in layout design.

## 4. Conclusion

Based on the theory of cognitive psychology, according to Wickens information processing model, the cognitive model of human-machine interaction interface was established. Considering people's information organization law, visual search law, user memory characteristics, and so on, the human-machine interaction interface should conform to operators' cognitive ability for interface information. Based on the cognitive model, the layout principles of human-machine interaction interface were summarized as the layout constraints. The problem of solving layout scheme was transformed into combinatorial optimization problem, and GA-ACA was put forward to solve the problem, which realized the algorithmization of artificial optimization process.

Taking the layout of manipulators as example, the objective function of layout optimization was built according to each principle. Use GA to generate the solution of layout scheme, which would be transformed into initial pheromone as ACA required. Taking the difference value of comprehensive weight of the manipulators for the layout principles as heuristic information of ACA, combined with the pheromone provided by GA, the positive feedback optimization mechanism of ACA was used to solve further. GA-ACA integrated the complementary advantages of GA and ACA, which had good optimizing performance and time performance and improved the design efficiency. Taking the 16 manipulators of ZJ120/9000DB rig console as layout example, layout design system of human-computer interaction interface for cabin was developed by Visual Basic, which validated the above layout optimization method.

## Figures and Tables

**Table 1:** Partition coding for manipulators.

	The name of the manipulator	Code		The name of the manipulator	Code
Range 1	E-BRAKE	1	Range 3	LEFT CATHEAD	9
	GEN E-STOP	2		RIGHT CATHEAD	10
	VFD E-STOP	3		ROTARY CATHEAD	11
	PARKING BRAKE	4		ALARM	12

Range 2	RT MOTOR CONTROL	5	Range 4	AIR SPINNER	13
	RT INERTIA BRAKE	6		TOOLS SELECT	14
	RT SPEED SETTING	7		SPARE1	15
	RT TORQUE LIMIT	8		SPARE2	16

**Table 2:** The size of each type of manipulators (mm).

The types of manipulators	Size	The codes of manipulators
Switch knobs	Φ30	4, 5, 6, 9, 10, 11, 12, 13, 14, 15, 16
Rotary knobs	Φ50	7, 8
Buttons	Φ40	1, 2, 3

**Table 3:** The manipulators' weight relative to Principles 2 and 3.

	1	2	3	4	5	6	7	8	9	10	11	12	13	14	15	16
*T*_*i*_	0.018	0.018	0.018	0.055	0.113	0.076	0.205	0.163	0.068	0.068	0.068	0.016	0.016	0.098	0	0
*S*_*i*_	0.163	0.115	0.115	0.115	0.069	0.069	0.069	0.069	0.043	0.043	0.043	0.013	0.026	0.026	0.011	0.011

**Table 4:** The correlation between the 16 manipulators.

*O*_*ij*_	1	2	3	4	5	6	7	8	9	10	11	12	13	14	15	16
1	1	0.5	0.7	0.9	0	0	0	0	0	0	0	0	0	0	0	0
2	0.5	1	0.9	0.5	0	0	0	0	0	0	0	0	0	0	0	0
3	0.7	0.9	1	0.7	0	0	0	0	0	0	0	0	0	0	0	0
4	0.9	0.5	0.7	1	0	0	0	0	0	0	0	0	0	0	0	0
5	0	0	0	0	1	0.9	0.7	0.9	0	0	0	0	0	0	0	0
6	0	0	0	0	0.9	1	0.5	0.7	0	0	0	0	0	0	0	0
7	0	0	0	0	0.7	0.5	1	0.9	0	0	0	0	0	0	0	0
8	0	0	0	0	0.9	0.7	0.9	1	0	0	0	0	0	0	0	0
9	0	0	0	0	0	0	0	0	1	0.9	0.7	0.5	0	0	0	0
10	0	0	0	0	0	0	0	0	0.9	1	0.7	0.5	0	0	0	0
11	0	0	0	0	0	0	0	0	0.7	0.7	1	0.5	0	0	0	0
12	0	0	0	0	0	0	0	0	0.5	0.5	0.5	1	0	0	0	0
13	0	0	0	0	0	0	0	0	0	0	0	0	1	0.9	0.3	0.3
14	0	0	0	0	0	0	0	0	0	0	0	0	0.9	1	0.3	0.3
15	0	0	0	0	0	0	0	0	0	0	0	0	0.3	0.3	1	0.9
16	0	0	0	0	0	0	0	0	0	0	0	0	0.3	0.3	0.9	1

**Table 5:** The comparison between GA, ACA, and ACA.

Algorithm	Layout scheme	The value of layout scheme	Number of iterations
GA	4, 2, 3, 1, 7, 8, 6, 5, 12, 11, 9, 10, 13, 14, 15, 16	2.814	82
ACA	4, 1, 2, 3, 5, 6, 7, 8, 9, 10, 11, 12, 15, 16, 14, 13	2.773	75
GA-ACA	4, 1, 2, 3, 7, 8, 5, 6, 12, 9, 10, 11, 15, 16, 13, 14	2.835	50 + 13

## References

[1] Cha J. Z., Tang X. J., Lu Y. P. (2002). Survey on packing problems. *Journal of Computer-Aided Design & Computer Graphics*.

[2] Dowsland K. A., Dowsland W. B. (1992). Packing problems. *European Journal of Operational Research*.

[3] Margaritis S., Marmaras N. (2007). Supporting the design of office layout meeting ergonomics requirements. *Applied Ergonomics*.

[4] Shariatzadeh N., Sivard G., Chen D. Software evaluation criteria for rapid factory layout planning, design and simulation.

[5] Huo J. Z., Li G. Q., Teng H. F., Sun Z. G. (2005). Human-computer cooperative ant colony/genetic algorithm for satellite module layout design. *Chinese Journal of Mechanical Engineering*.

[6] Li R., Zhuang D. M., Wang R., Wang L. R. (2004). Optimization about the layout work of the steering arrangement in the cockpit. *Journal of System Simulation*.

[7] Yan S. Y., Chen Y., Liang L. Y. (2014). Optimization of controls layout based on simulated annealing algorithm. *Nuclear Power Engineering*.

[8] Zong L. C., Ye C., Yu S. H., Chen D. K. (2013). Research and application of intelligent layout method in DSV cabin equipment. *Shipbuilding of China*.

[9] Zong L. C., Yu S. H., Sun J. B., Han L. W., An S. S. (2014). Study on cabin layout optimization with fish algorithm. *Mechanical Science and Technology for Aerospace Engineering*.

[10] Wang Y. L., Wang C., Ji Z. S., Zhao X. G. (2013). A study on intelligent layout design of ship cabin. *Shipbuilding of China*.

[11] Wolpert D. H., Macready W. G. (1997). No free lunch theorems for optimization. *IEEE Transactions on Evolutionary Computation*.

[12] Zhao F. Q., Li G. Q., Yang C., Abraham A., Liu H. B. (2014). A human-computer cooperative particle swarm optimization based immune algorithm for layout design. *Neurocomputing*.

[13] Lodi A., Martello S., Monaci M. (2002). Two-dimensional packing problems: a survey. *European Journal of Operational Research*.

[14] Anderson J. R. (2012). *Cognitive Psychology and It's Implications*.

[15] Ghanbari A., Kazemi S. M. R., Mehmanpazir F., Nakhostin M. M. (2013). A cooperative ant colony optimization-genetic algorithm approach for construction of energy demand forecasting knowledge-based expert systems. *Knowledge-Based Systems*.

[16] Best J. B. (2000). *Cognitive Psychology*.

[17] Wickens C., Hollands J. G., Banbury S., Parasuraman R. (2012). *Engineering Psychology & Human Performance*.

[18] Sun L. Y., Li Z. X., Jin T. S. (1997). Cognitive synthetic model and its application in HCI. *Journal of Xi'an Jiaotong University*.

[19] Zhang Y. P. (2005). Human-computer interface design based on knowledge of cognitive psychology. *Computer Engineering and Applications*.

[20] Lan Z. (1998). The cognitive basis of software interface design. *Shanxi Science and Technology*.

[21] Wang X. P., Cao L. M. (2002). *Genetic Algorithm—Theory, Application and Software Implementation*.

[22] Bonabeau E., Dorigo M., Theraulaz G. (2000). Inspiration for optimization from social insect behaviour. *Nature*.

[23] Dorigo M., Gambardella L. M. (1997). Ant colonies for the travelling salesman problem. *BioSystems*.

[24] Dorigo M., Maniezzo V., Colorni A. (1996). Ant system: optimization by a colony of cooperating agents. *IEEE Transactions on Systems, Man, and Cybernetics, Part B: Cybernetics*.

[25] Ding J.-L., Chen Z.-Q., Yuan Z.-Z. (2004). On the Markov convergence analysis for the combination of genetic algorithm and ant algorithm. *Acta Automatica Sinica*.

[26] Lee Z.-J., Su S.-F., Chuang C.-C., Liu K.-H. (2008). Genetic algorithm with ant colony optimization (GA-ACO) for multiple sequence alignment. *Applied Soft Computing*.

[27] Li Z., Xu C. P., Mo W., Chen G. J. (2003). Initialization for synchronous sequential circuits based on ant algorithm & genetic algorithm. *Acta Electronica Sinica*.

[28] Xiong Z.-H., Li S.-K., Chen J.-H. (2005). Hardware/software partitioning based on dynamic combination of genetic algorithm and ant algorithm. *Journal of Software*.

[29] Ding J.-L., Chen Z.-Q., Yuan Z.-Z. (2003). On the combination of genetic algorithm and ant algorithm. *Journal of Computer Research and Development*.

[30] Stützle T., Hoos H. H. (2000). MAX-MIN ant system. *Future Generation Computer Systems*.

[31] Stuetzle T., Hoos H. MAX-MIN Ant System and local search for the traveling salesman problem.

